# Supplementary material for: Sorting of secretory proteins at the trans-Golgi network by human TGN46
Source: eLife. 2024 Mar 11;12:RP91708. doi: 10.7554/eLife.91708 (PMC10928510; doi:10.7554/eLife.91708)

Uncropped gel image of Figure 1A

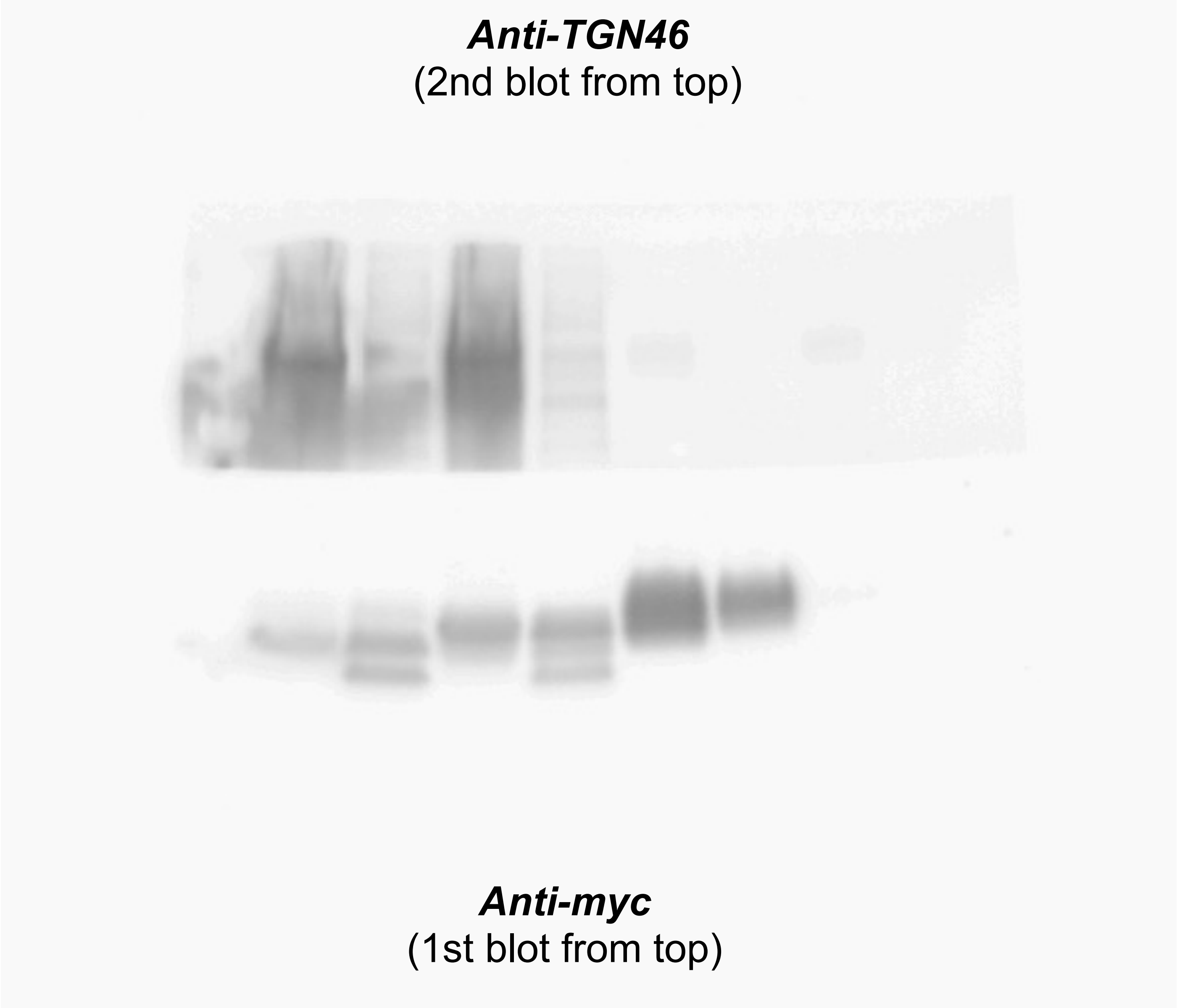

Uncropped gel image of Figure 1A

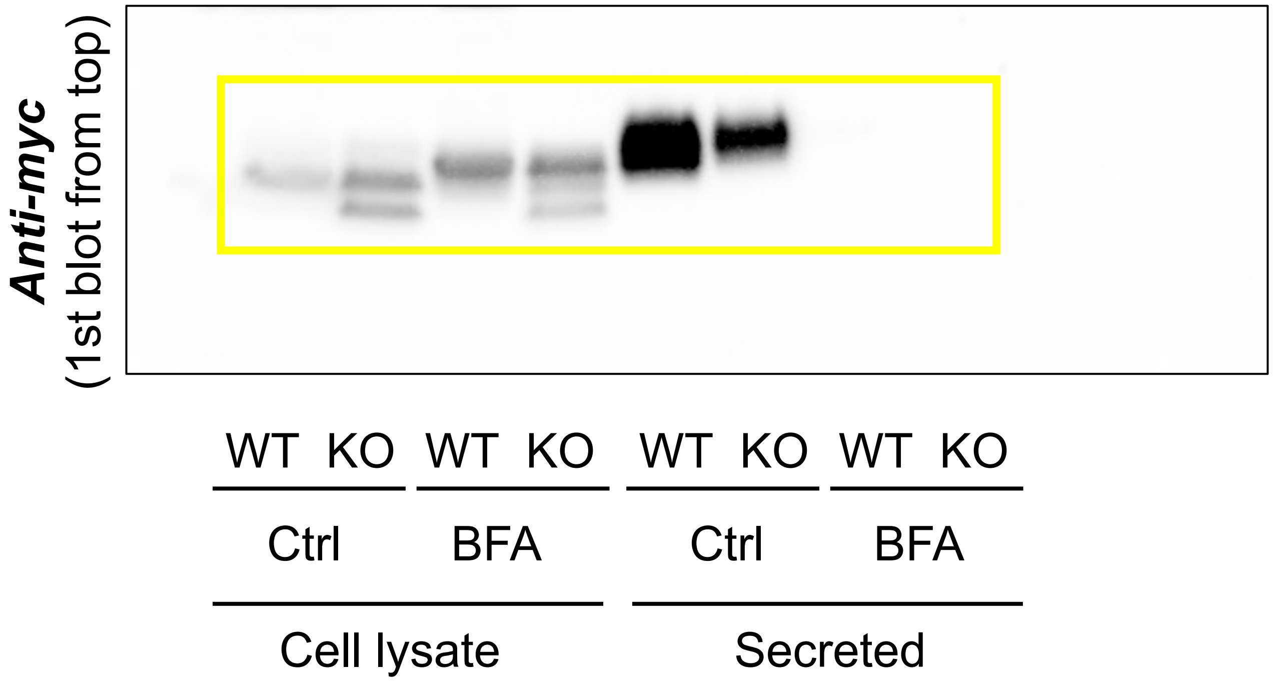

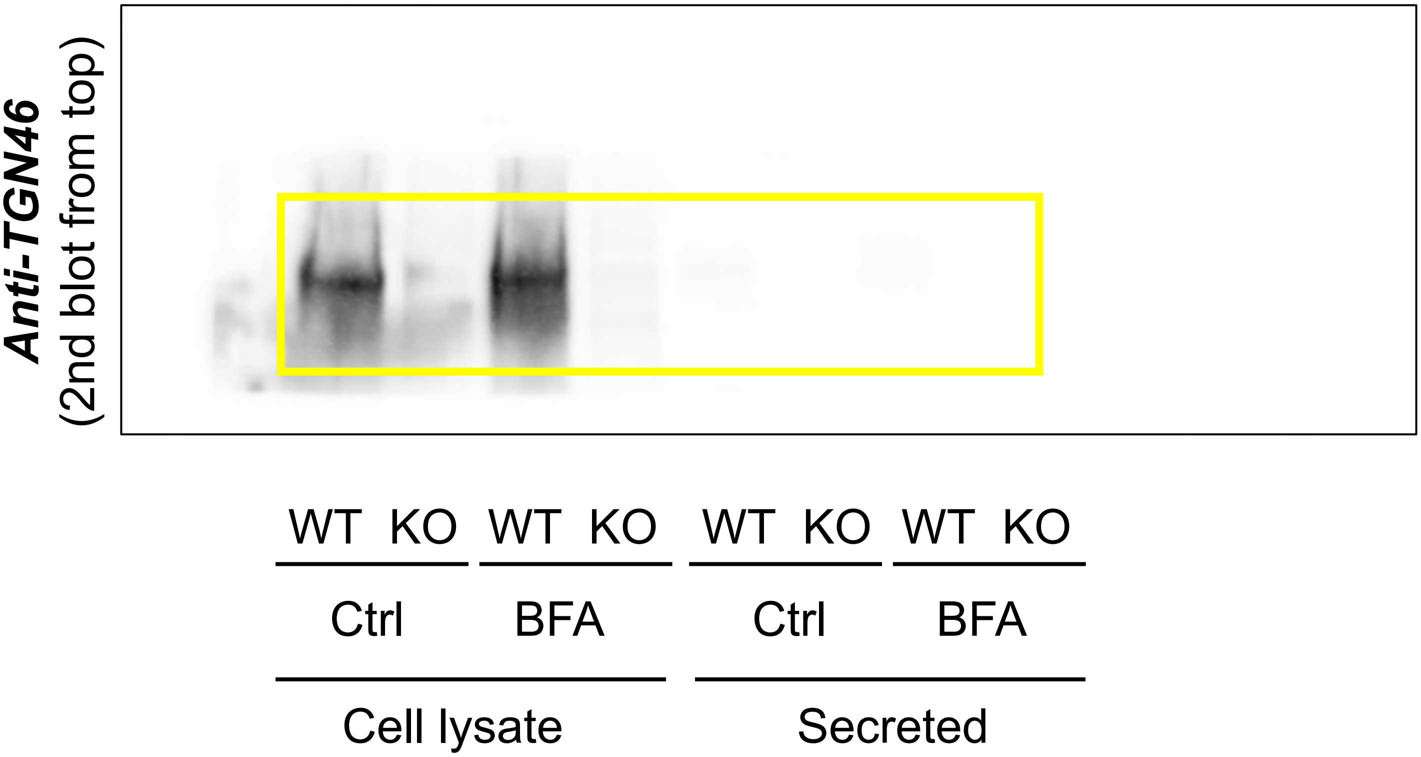

Supplement: Figure 1—source data 1. [file elife-91708-fig1-data1.zip › Fig1A-WB/Fig1A-source.pdf]
