# Supplementary material for: Sorting of secretory proteins at the trans-Golgi network by human TGN46
Source: eLife. 2024 Mar 11;12:RP91708. doi: 10.7554/eLife.91708 (PMC10928510; doi:10.7554/eLife.91708)
Supplement: Figure 1—source data 1. [file elife-91708-fig1-data1.zip › Fig1A-WB/Fig1A-source.pptx]

## Slide 1
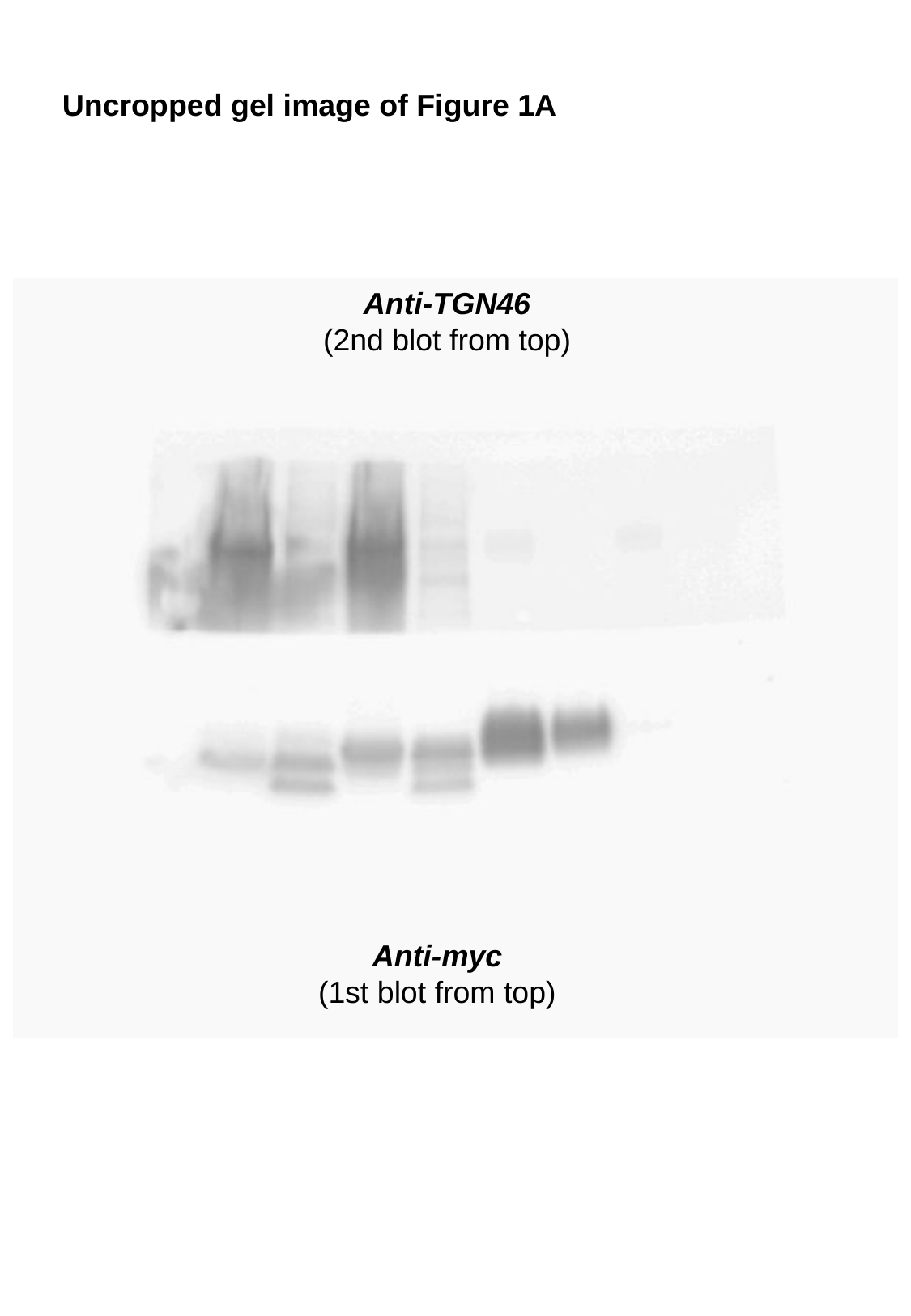

Uncropped gel image of Figure 1A
Anti-TGN46
(2nd blot from top)
Anti-myc
(1st blot from top)

## Slide 2
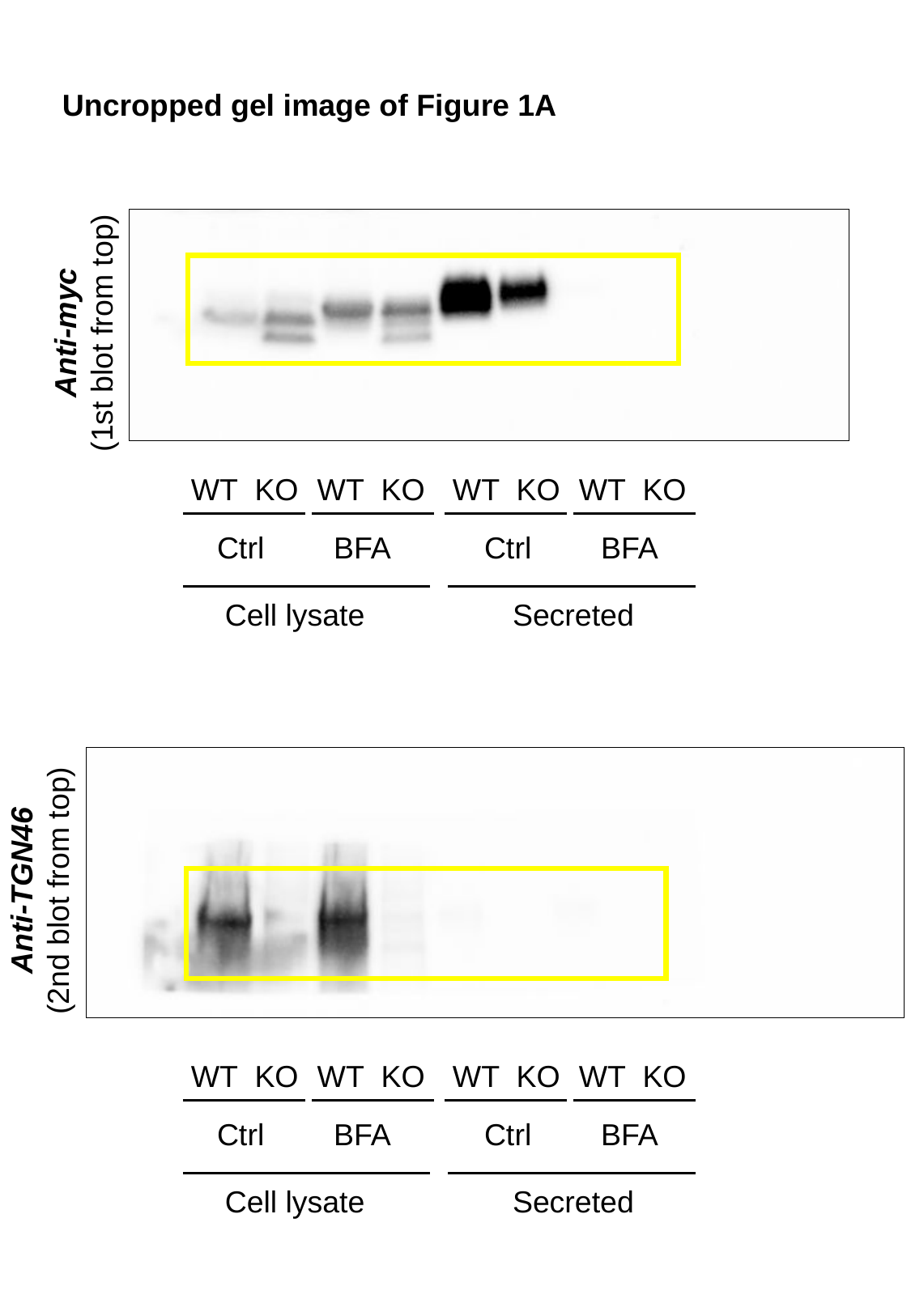

Uncropped gel image of Figure 1A
Anti-myc
(1st blot from top)
WT
KO
WT
KO
WT
KO
WT
KO
Ctrl
BFA
Ctrl
BFA
Cell lysate
Secreted
Anti-TGN46
(2nd blot from top)
WT
KO
WT
KO
WT
KO
WT
KO
Ctrl
BFA
Ctrl
BFA
Cell lysate
Secreted
