# Supplementary material for: Sorting of secretory proteins at the trans-Golgi network by human TGN46
Source: eLife. 2024 Mar 11;12:RP91708. doi: 10.7554/eLife.91708 (PMC10928510; doi:10.7554/eLife.91708)
Supplement: Figure 4—figure supplement 3—source data 1. [file elife-91708-fig4-figsupp3-data1.zip › FigS5C-WB/FigS5C-Source.pptx]

## Slide 1
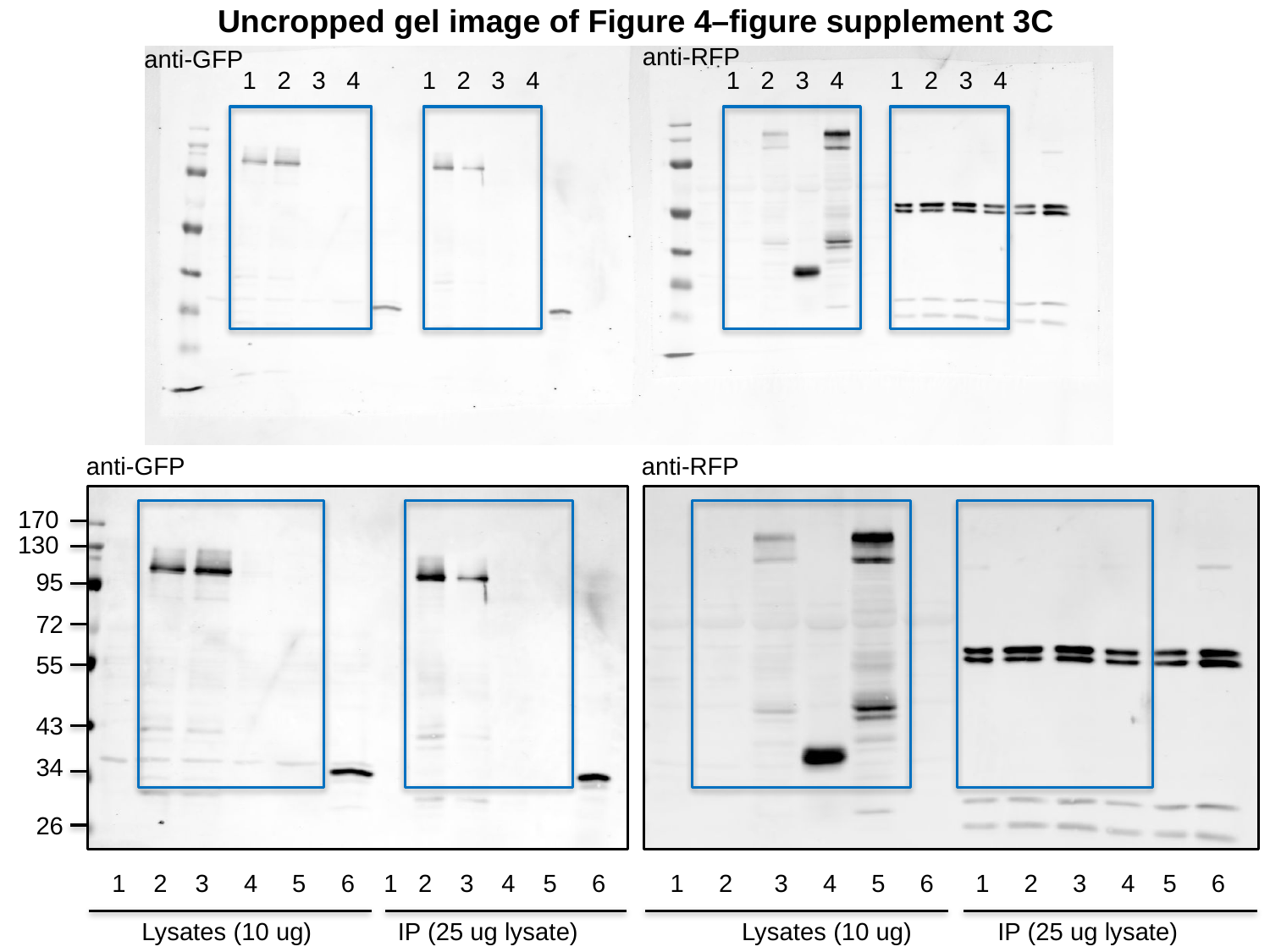

Uncropped gel image of Figure 4–figure supplement 3C
anti-RFP
anti-GFP
1 2 3 4
1 2 3 4
1 2 3 4
1 2 3 4
anti-GFP
anti-RFP
170
130
95
72
55
43
34
26
1 2 3 4 5 6
1 2 3 4 5 6
1 2 3 4 5 6
1 2 3 4 5 6
Lysates (10 ug)
IP (25 ug lysate)
Lysates (10 ug)
IP (25 ug lysate)
