# Supplementary figures and images for: Sorting of secretory proteins at the trans-Golgi network by human TGN46
Source: eLife. 2024 Mar 11;12:RP91708. doi: 10.7554/eLife.91708 (PMC10928510; doi:10.7554/eLife.91708)

# Uncropped gel image of Figure 4—figure supplement 3C

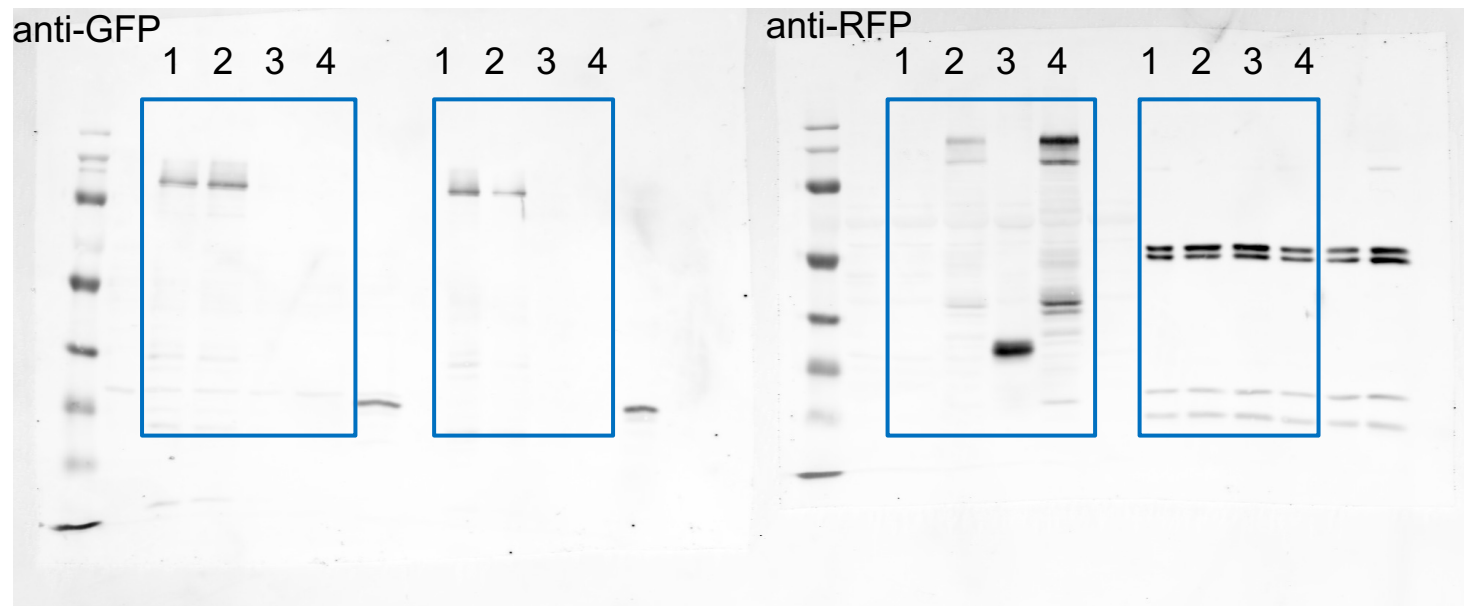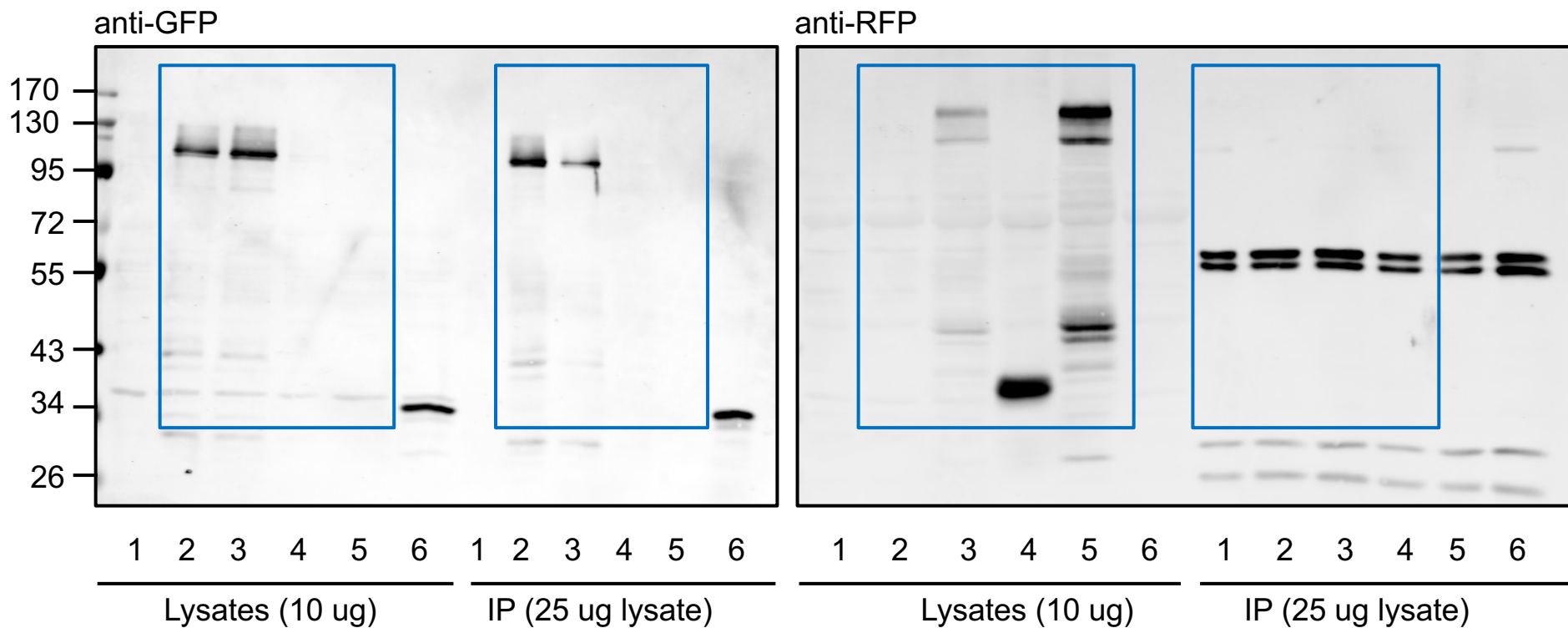

Supplement: Figure 4—figure supplement 3—source data 1. [file elife-91708-fig4-figsupp3-data1.zip › FigS5C-WB/FigS5C-Source.pdf]
